# Medical Colleges

**Published:** 1848-10

**Authors:** 


### MEDICAL COLLEGES.

In our last number, we gave a tabular statement of the number of Professors and length of the lecture term in many of the Medical Colleges in the United States. The list was necessarily incomplete, from the imperfect data in our possession; and later publications of several of them enable us now to correct some errors in our statement.

Thus, in the Winchester College it was said the term lasted *seven* months; it should have been *eight* months.

The term in the College of Physicians and Surgeons of New York was put down as *five months and a half*, instead of *five months*.

We have in addition to those included in our former statement:

The Pennsylvania Medical College, six Professors—length of term, four and a half months.

Franklin Medical College of Philadelphia, six Professors—commences 16th of October; duration of term not stated.

National Medical College, Washington, D. C., seven Professors, one Adjunct and one Associate—length of term, five months.

The Southern Medical and Surgical Journal says: "No circular that we have seen has remained silent on the subject of reform, and although *all* the Institutions have not come up to the recommendations of the Convention, some have done so fully, and many others partially." (Sept. 1848.) Will the editor have the goodness to mention the names of those Institutions which have come up to the recommendations of the Convention "*fully*"? We have been somewhat attentive to this subject, and we do not know of *one* instance of the kind.

